# Effectiveness of a Smartphone App (MINISTOP 2.0) integrated in primary child health care to promote healthy diet and physical activity behaviors and prevent obesity in preschool-aged children: randomized controlled trial

**DOI:** 10.1186/s12966-023-01405-5

**Published:** 2023-02-21

**Authors:** Christina Alexandrou, Hanna Henriksson, Maria Henström, Pontus Henriksson, Christine Delisle Nyström, Marcus Bendtsen, Marie Löf

**Affiliations:** 1 Department of Health, Medicine and Caring Sciences, Division of Society and Health, Linköping University, 581 83 Linköping, Sweden; 2 Department of Biosciences and Nutrition, Karolinska Institutet, NEO, Huddinge, 141 83 Sweden

**Keywords:** mHealth, Primary child health care, Early prevention, Preschool, Diet, Physical activity, Childhood overweight and obesity, Smartphone app, Randomized controlled trial

## Abstract

**Background:**

Childhood overweight and obesity is a public health priority. We have previously reported the efficacy of a parent-oriented mobile health (mHealth) app-based intervention (MINISTOP 1.0) which showed improvements in healthy lifestyle behaviors. However, the effectiveness of the MINISTOP app in real-world conditions needs to be established.

**Objective:**

To evaluate the real-world effectiveness of a 6-month mHealth intervention (MINISTOP 2.0 app) on children’s intake of fruits, vegetables, sweet and savory treats, sweet drinks, moderate-to-vigorous physical activity, and screen time (primary outcomes), and on parental self-efficacy (PSE) for promoting healthy lifestyle behaviors, and children’s body mass index (BMI) (secondary outcomes).

**Methods:**

A hybrid type 1 effectiveness-implementation design was utilized. For the effectiveness outcomes, a two-arm, individually randomized controlled trial was conducted. Parents (*n* = 552) of 2.5-to-3-year-old children were recruited from 19 child health care centers across Sweden, and, randomized to either a control (standard care) or intervention group (MINISTOP 2.0 app). The 2.0 version was adapted and translated into English, Somali and Arabic to increase reach. All recruitment and data collection were conducted by the nurses. Outcomes were assessed at baseline and after six months, using standardized measures (BMI) and a questionnaire (health behaviors, PSE).

**Results:**

Among the participating parents (*n* = 552, age: 34.1 ± 5.0 years), 79% were mothers and 62% had a university degree. Twenty-four percent (*n* = 132) of children had two foreign-born parents. At follow-up, parents in the intervention group reported lower intakes of sweet and savory treats (-6.97 g/day; *p* = 0.001), sweet drinks (-31.52 g/day; *p* < 0.001), and screen time (-7.00 min/day; *p* = 0.012) in their children compared to the control group. The intervention group reported higher total PSE (0.91; *p* = 0.006), PSE for promoting healthy diet (0.34; *p* = 0.008) and PSE for promoting physical activity behaviors (0.31; *p* = 0.009) compared to controls. No statistically significant effect was observed for children’s BMI z-score. Overall, parents reported high satisfaction with the app, and 54% reported using the app at least once a week.

**Conclusion:**

Children in the intervention group had lower intakes of sweet and savory treats, sweet drinks, less screen time (primary outcomes) and their parents reported higher PSE for promoting healthy lifestyle behaviors. Our results from this real-world effectiveness trial support the implementation of the MINISTOP 2.0 app within Swedish child health care.

**Trial registration:**

Clinicaltrials.gov NCT04147039; https://clinicaltrials.gov/ct2/show/NCT04147039

**Supplementary Information:**

The online version contains supplementary material available at 10.1186/s12966-023-01405-5.

## Background

Globally, childhood overweight and obesity is expected to increase by 60%, reaching an estimated 250 million children in 2030 [1, 2]. In Sweden, the prevalence of overweight and obesity in children has been reported to be 11% in 4-year-olds and 21% in 6–9-year-olds, indicating a prevalence almost twice as high in school-aged compared to preschool-aged children [3, 4]. Childhood overweight and obesity tends to persist into adulthood [5, 6], and is associated with impaired psychosocial and cardiometabolic health [2]. Furthermore, there is an uneven distribution of obesity, with a higher prevalence in socioeconomically disadvantaged areas in high-income countries, including Sweden [7, 8]. In terms of healthy lifestyle behaviors such as intake of fruit and vegetables and physical activity, only 20% and 30% of Swedish preschool-aged children reach the national recommendations respectively [9, 10]. Clearly, population level efforts to promote healthy lifestyle behaviors and to counteract early onset of overweight and obesity is a public health priority, with primary child health care identified as a key setting [11].

Previous child health care-based interventions have shown limited effectiveness in terms of obesity prevention and promotion of healthy lifestyle behaviors [11–15], indicating a need for additional efforts exploring new types of interventions. Digital health technologies have been reported to improve health care quality through increased access to health information and services [16]. Also, the use of mobile phone based (mHealth) programs to deliver lifestyle or weight loss interventions in adults, older children and adolescents have shown to facilitate health behavior change and weight loss [17–22]. mHealth technology enables adaptations and upscaling of interventions to fit larger population groups [16]. It also provides opportunity for increased family engagement connected to the health care system as well as support in between visits. Thus, using mHealth to deliver obesity prevention interventions is in line with both the WHO’s and the Swedish government’s guidelines, where health care systems should aspire to become increasingly responsive to individuals’ needs, e.g., through digital solutions that support family and self-managed care [16, 23].

We have previously developed a novel mHealth intervention, the MINISTOP application (app), aimed at preventing onset of overweight and obesity in preschool-aged children, by supporting parents in promoting healthy lifestyle behaviors [24]. The efficacy of the MINISTOP app was evaluated in a randomized controlled trial using accurate and objective outcome measures such as air-displacement plethysmography using the pediatric option for BodPod [25] for measuring body composition (i.e., fat mass index) and accelerometry (ActiGraph wGT3X-BT) for measuring physical activity [24]. We observed no group difference in fat mass index, but the intervention group demonstrated a statistically significant higher composite score of six dietary and physical activity behaviors at follow-up (OR: 1.99; 95% CI 1.20–3.30; *p* = 0.008). This effect was also more pronounced in children with a higher fat mass index [24]. Noteworthy, the trial reported a similar effect size as more traditional labor intense face-to-face interventions [14, 15]. However, the app was distributed by researchers and not by health care professionals (i.e., real-world conditions). For large-scale implementation in routine care, real-world effectiveness should also be proven. Also, as this first version of the app was available only in Swedish, it was not accessible to parents who did not speak Swedish. To date, it is estimated that 24% of children in Sweden have parents with a foreign background [26]; the majority being from Syria, Somalia, and Iraq [27]. Thus, to also reach families with diverse backgrounds, an adapted and translated (Somali, Arabic, and English) version of the app, MINISTOP 2.0, was developed for evaluation within child health care settings. The adaptations made were based on our findings from interviews with Somali-, Arabic- and Swedish speaking parents (end-users) and with child health care nurses (future implementers) [28].

### Aim

As reported in our study protocol [29], we have designed a hybrid type 1 effectiveness-implementation trial [30] to simultaneously evaluate effectiveness as well as implementation aspects of the MINISTOP 2.0 app. The effectiveness is evaluated through an individually randomized controlled trial in 2.5–3-year-old children [29]. In this paper we report the effectiveness results, while the implementation outcomes will be reported elsewhere. Thus, the specific aims for this paper were to evaluate the effectiveness of a 6-month parent-oriented mHealth intervention (MINISTOP 2.0 app), embedded in the routine services of Swedish primary child health care, on:

1. children’s intake of fruits, vegetables, sweet and savory treats, sweet drinks, and time spent in moderate-to-vigorous physical activity (MVPA) and screen time (primary outcomes).
2. parental self-efficacy (PSE) for promoting healthy dietary, physical activity and screen time behaviors in children, and children’s body mass index (BMI) (secondary outcomes).

## Methods

### Study design

The MINISTOP 2.0 is a hybrid type 1 trial evaluating effectiveness and implementation outcomes [29]. For effectiveness, a two-arm parallel group individually randomized controlled trial was conducted. The trial was conducted between the 2^nd^ of November 2019 and the 8^th^ of April 2022 within Swedish primary child health care. The study design and analysis plan has been described in detail in the study protocol [29]. The implementation outcomes will be reported separately. The MINISTOP 2.0 trial was approved by the Swedish Ethical Review Authority (ref no 2019–02747; 2020–01526). Written informed consent was collected from all participating parents prior to baseline assessments. The trial is reported according to the Consolidated Standards of Reporting Trials (CONSORT) guidelines (Additional file 1) [31] and the TIDieR Checklist Additional file 2 [32].

### Participants and baseline procedures

Parents with a 2.5–3-year-old child were recruited during a routine visit to primary child health care. Recruitment took place in 19 child health care centers covering diverse geographic and socioeconomic areas in six Swedish regions (Skåne, Stockholm, Uppsala, Västmanland, Västra Götaland and, Östergötland). All recruitment procedures, randomization, and data collection were conducted by the child health care nurses at each study site. Written information about the trial was included in the invitation for the routine visit that was mailed to the home address of all eligible parents. Parents were eligible if they were able to read and/or speak Swedish, Somali, Arabic or English sufficiently well to understand the study information and provide informed consent. Families were not included if the child was diagnosed with a neurological or endocrine disease or if one parent was suffering from a serious physical or mental illness, as participation would be too demanding. During the visit, nurses reminded parents about the study invitation and provided additional verbal information about the study if requested. Thereafter, parents who consented to participate completed a paper questionnaire regarding their child’s diet, MVPA, screen time, physical fitness [33], and dental health behaviors. The questionnaire also included questions about PSE for promoting healthy dietary, physical activity and screen time behaviors [34] as well as background questions about level of education and country of birth (parents and child). The child’s height and weight were then measured by the nurse, using standardized procedures. The research group conducted a start-up training session at each child health care center prior to study start, and then collected all data for statistical analyses after the completion of all follow-up visits.

### Randomization, blinding, and follow-up

Following baseline assessments, nurses used opaque envelopes for randomization. These were created using a random allocation sequence generated in R 3.6.1 for each study site using block randomization (with random block sizes of 2 and 4) [29]. As nurses provided access to the MINISTOP 2.0 app to the parents in the intervention group through a caregiver interface, neither were blinded to the group allocation. After the 6-month intervention period, parents were invited to a follow-up visit where nurses again measured the child’s height and weight and parents filled in the same questionnaire as at baseline. At this visit, parents in the intervention group were also asked to rate their satisfaction with the MINISTOP 2.0 app by means of a questionnaire.

### Control group

The control group received the standard care offered by the Swedish primary child health care system during the routine visit at 2.5/3-years [35]. This includes a conversation about healthy foods and eating behaviors and health behaviors in general, as well as a pamphlet with information on healthy lifestyle behaviors.

### Intervention

**Fig. 1:**
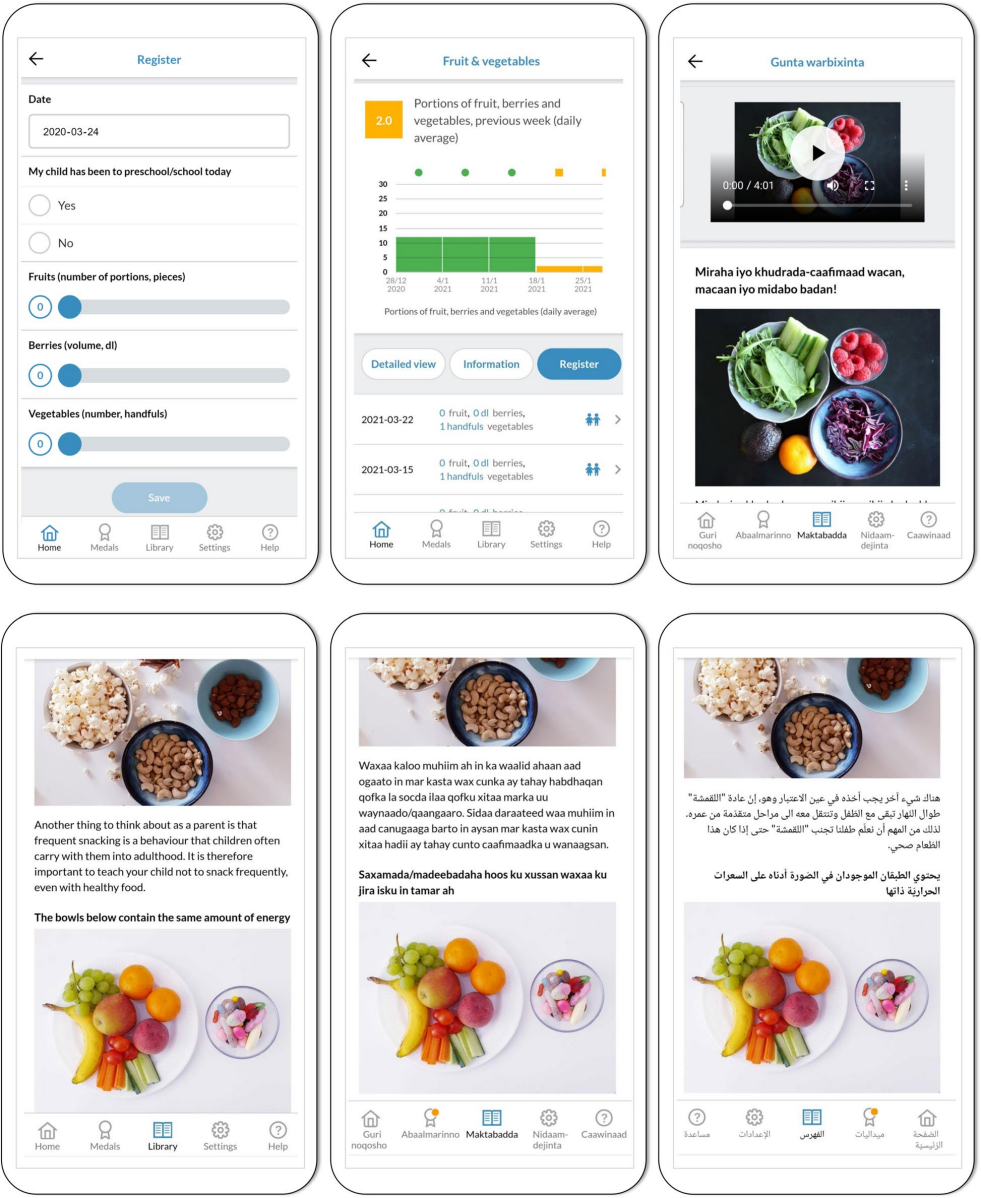
Screenshots from the MINISTOP 2.0 app showing some of its key features. From the upper left corner: 1) the registration feature for daily intake of fruits, berries, and vegetables, 2) an example of the graphical output of the weekly registrations summarized at the end of each week, 3) an example of the audio–video feature in the Somali app-version, and 4–6) an example of a theme text in different languages (English, Somali, and Arabic)

Participants in the intervention group were, in addition to standard care, given immediate access to the MINISTOP 2.0 app, which is a digital intervention delivered via a smartphone application (both iOS and Android compatible) aimed at supporting parents in promoting health behaviors in their children. The app is available in four different languages: Swedish, Somali, Arabic and English. The intervention builds on the MINISTOP 1.0 digital platform and content [24] and delivers an extensive program of information, grounded in social cognitive theory [36] and key behavior change techniques [37] such as shaping knowledge, goal setting, identification of barriers, self-monitoring of behavior, and feedback, over a period of 6 months (Supplementary information, Figure S1). The intervention content is based on evidence-based recommendations on diet [38], physical activity and screen time [39, 40] for preschool-aged children, divided into 13 themes, where a new theme is released every two weeks. The themes are: 1) healthy everyday food, 2) healthy breakfast, 3) healthy snacks, 4) physical activity and screen time, 5) sweets and snacks, 6) fruit and vegetables, 7) beverages, 8) snacking, 9) fast food, 10) sleep, 11) meals outside the home, 12) foods as a reward/on special occasions and 13) dental health. To make it easier for the users to navigate the information in the app, each theme is divided into three parts: general information, practical tips, and strategies. The app also includes a feature where parents can enter their child’s intake of fruits and vegetables, sweets, snacks, sugar sweetened drinks as well as time spent being physically active and screen time. At the end of each week the app delivers feedback (graphical and text message) based on the entries. Screenshots of the main features of the MINISTOP 2.0 app are shown in Fig. 1. As mentioned earlier, the MINISTOP app was piloted among parents and nurses prior to study start in a series of qualitative interviews [28]. Findings from the interviews helped us further improve and adapt the content and features of the app before translation to Somali, Arabic and English. One of the main additions to the 2.0 version of the app was an audio/video-feature of the theme content (themes 1–13) to make it more accessible and facilitate participation regardless of literacy. Examples of further additions were videos on key strategies for healthy eating behaviors and pictures illustrating e.g., food portion sizes, the maximum weekly intake of sweets, and recommendations of fruits and vegetables for 2-to-3-year-old children, along with attainable tips and strategies on how to reach these recommendations. Furthermore, certain cultural adaptations were made, such as addition of domestic and relatable food items to some of the food and snack recipes. Parts of the text e.g., referring to specific Swedish traditions that may not be relatable for parents from other cultures were also reworked. Finally, when adding images for illustrating the information in the app we strived for representation. Thus, the app includes images of children and families from many different countries and cultures, to increase the feeling of inclusion and recognition.

### Outcomes

The primary outcomes were parent-reported children’s intake of fruits, vegetables, sweet and savory treats, sweet drinks, as well as time spent in MVPA and screen time, after having completed the 6-month intervention. Secondary outcomes were children’s BMI and PSE for supporting healthy lifestyle behaviors (healthy diet, adequate physical activity, limiting screen time) in their children.

### Health behaviors

As aforementioned, our first evaluation of the MINISTOP app showed a positive effect on a composite score based on dietary and physical activity variables assessed using accurate and objective methodologies [24]. Here in the second phase, the primary aim was to evaluate effectiveness in a real-world setting with data being collected by child health care nurses. Thus, all study assessments had to be feasible, i.e., easily administrated, and possible to complete within the timeframe of the routine visit at the child health care center. Therefore, a short questionnaire was used for all outcome measures at both baseline and follow-up. The questionnaire included validated questions [33] on diet and physical activity used by the Swedish National Board of Health and Welfare [41], that were modified to fit 2.5–3-year-old children [29]. The dietary questions included consumption of key dietary indicators such as vegetables, fruits and berries, sweet and savory treats, as well as sweet drinks, assessed as the number of average standardized portions per day during the past month. The questions on dietary indicators were worded as follows: *“How many portions of fruits or berries (fresh, frozen, tinned, *etc*.) does your child eat per day? One portion equals 1 normal size fruit or 1 dl of berries or fruit pieces. Think back over the past month”.* The number of standardized portions per day reported were then converted into grams per day using standardized weights from the Swedish Food Agency’s food database [42]. Correspondingly, parents reported their child’s average MVPA and screen time [29] in minutes per day separately on weekdays and weekend days. The questions assessing MVPA were worded as follows: *“On a normal weekday, how much time does your child spend doing physical activity that causes their heart to beat faster and sometimes makes them out of breath? Think back over the past month”.* A weighted average of time spent in MVPA on weekdays and weekend days was then used in the analyses: (MVPA weekday * 5) + (MVPA weekend day * 2) divided by 7. A corresponding weighted average for screen time was calculated.

### BMI

Children’s height and weight were assessed by their nurse at baseline and follow-up using standardized procedures. Children were weighed in light clothing without shoes and height was measured using a wall-mounted stadiometer. BMI was calculated as weight divided by height squared (kg/m^2^). The extended international age and sex specific body mass index (IOTF) cut-offs by Cole and Lobstein were used for classification of children’s BMI into weight status categories (i.e., underweight, normal weight, overweight and obesity), and for calculation of BMI standard deviation scores (BMI z-scores) [43].

### Parental self-efficacy

PSE for promoting healthy lifestyle behaviors was assessed using questions from the previously validated Parental Self-Efficacy for Promoting Healthy Physical Activity and Dietary Behaviors in Children Scale (PSEPAD) Questionnaire [34]. As reported in the study protocol [29], the questions covered PSE for a) promoting healthy dietary behaviors, b) promoting healthy physical activity and, c) limiting screen time in their children. In each question, parents were asked to rate their self-efficacy for the respective lifestyle behavior on a scale from 0–10, where 0 corresponded to the lowest and 10 to the highest perceived self-efficacy (0:”Not at all”; 1–2: “To a very low extent; 3–4: “To some extent”; 5–6: “To quite an extent”; 7–8: “To a high extent” and 9–10: “To a very high extent”). For the analyses, a total PSE score was created in addition to the PSE scores for each separate health behavior. The maximum possible score for total PSE was 30.

### Sample size

A minimum of 360 participants (180 in each group) would provide 80% power (α = 0.05) to detect a 0.30 standard deviation (SD) difference in outcomes between groups. This corresponds to e.g., a difference of 25 g in fruit and vegetable intake or a 0.4 kg/m^2^ difference in BMI. We set out to recruit at least 500 participants, to account for a maximum loss to follow-up and/or dropout rate of 25–30% given previous experiences [15, 24].

### Statistical analyses

All analyses followed the study protocol [29] and were intention-to-treat. Analyses were first conducted using complete cases, assuming that any missing data was missing completely at random (MCAR). We conducted attrition analyses to find evidence against the MCAR assumption and conducted sensitivity analyses with missing data imputed (using multiple imputations with chained equations [44]; 200 imputed data sets with 30 iterations).

Linear regression was used to contrast differences between the intervention and control group for all primary outcomes (vegetables and fruits, sweet and savory treats, sweet drinks, MVPA, and screen time) as well as the secondary outcome PSE. Group differences in the secondary outcome for BMI z-scores were analyzed using quantile regression (10^th^, 50^th^ and 90^th^ percentile). All regression models were adjusted for baseline values for each respective outcome, and the child’s sex and age at baseline. The models also included a random intercept for each child health care center to account for clustering of data points within centers. Interaction analyses were performed to investigate whether the effect of the intervention on primary and secondary outcomes differed depending on parental country of birth (both parents from Sweden, both parents from another country, or one parent from Sweden and one not) and parental level of education (primary school, highschool or university), where an interaction term between group allocation and country of birth and level of education respectively, was included. For null hypothesis testing a 0.05 level of significance (two-sided) was used. Additionally, we also conducted Bayesian analyses [45] to create a robust base for scientific inference for the effectiveness of the intervention. Statistical analyses were conducted in RStudio version 4.1.3 (The R Foundation for Statistical Computing).

## Results

### Baseline characteristics of participating children and parents

**Fig. 2:**
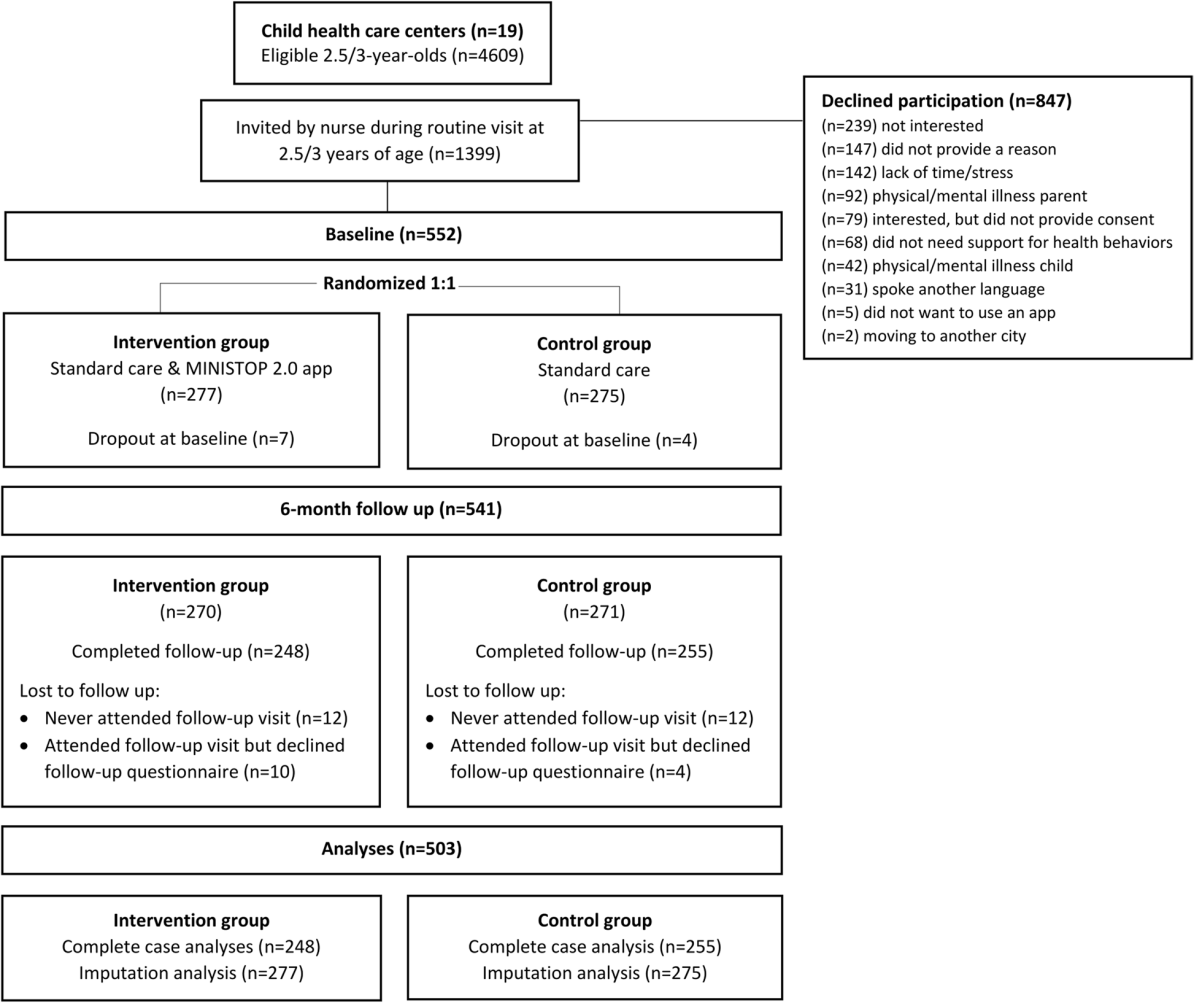
Flowchart of the recruitment and data collection in the MINISTOP 2.0 trial

A flowchart of the recruitment and data collection is presented in Fig. 2. Overall, approximately 4600 parents from 19 child health care centers attended a routine visit at 2.5 or 3-years during the recruitment period between November 2019 and September 2021. In total, 1399 families were invited and informed about the study. Out of these, 552 consented to participate, completed baseline assessments and were randomly assigned to the intervention (*n* = 277) or control group (*n* = 275). Baseline characteristics of the participating parents and children are shown in Table 1. Seventy-one percent of the participating parents were born in Sweden, 9% were born in another European country, and 20% were born outside of Europe. Most of the foreign-born parents were from North Africa and the Middle East (32%), Sub-Saharan Africa (19%) and Central Europe (17%). When considering the whole family, 24% of the children had two foreign-born parents. Among the children, 74% were classified as having normal weight, 16% with overweight, 5% with obesity and 6% with underweight. There were no differences between the intervention and control group in baseline characteristics except for the intervention group reporting a slightly higher intake of vegetables and lower intake of sweet and savory treats, however, all regression models were adjusted for the baseline outcome value. Attrition rates (Fig. 2) in both groups were low, with 6.9% and 5.8% lost to follow-up, i.e., never attended the follow-up visit, in the intervention and control group, respectively. Similarly, among the participants who came to the follow-up visit, only 3.6% and 1.5% had missing questionnaire data in the intervention and control group at follow-up, respectively.

**Table 1:** Baseline characteristics of participating parents and children (*n* = 552)

		All		Intervention		Control
	***N***	% or M (SD).^1^	***N***	% or M (SD)	***N***	% or M (SD)
**Parental characteristics**.^2^						
Age (years)	539	34.1 (5.0)	270	34.0 (4.9)	269	34.1 (5.1)
Female	428	78.8	218	80.4	210	77.2
Male	115	21.2	53	19.6	62	22.8
Education (%)						
Primary school (≤ 9 years)	27	5.0	15	5.6	12	4.4
Highschool (12 years)	177	32.6	81	30.0	96	35.3
University	338	62.4	174	64.4	164	60.3
Country of birth (%)						
Sweden	390	71.3	200	73.0	190	69.6
Europe	50	9.1	27	9.8	23	8.4
Outside of Europe	107	19.6	47	17.2	60	22.0
BMI (kg/m^2^)	532	25.5 (5.0)	265	25.5 (5.1)	267	25.5 (4.8)
PSE total score^3^	543	22.5 (4.6)	271	22.7 (4.8)	272	22.3 (4.3)
PSE diet	543	7.7 (1.7)	271	7.7 (1.8)	272	7.6 (1.6)
PSE physical activity	543	7.8 (1.7)	271	7.9 (1.7)	272	7.8 (1.7)
PSE screen time	543	7.0 (2.1)	271	7.2 (2.2)	272	6.9 (2.0)
**Children’s characteristics**						
Boys	279	50.5	131	47.3	148	53.8
Girls	273	49.5	146	52.7	127	46.2
2.5 years at baseline (%)	403	73.0	203	73.3	200	72.7
3.0 years at baseline (%)	149	27.0	74	26.7	75	27.3
Country of birth (%)^4^						
Sweden	535	97.4	270	97.8	265	97.1
Europe	6	1.1	3	1.1	3	1.1
Outside of Europe	8	1.5	3	1.1	5	1.8
BMI (kg/m^2^)	549	16.8 (1.5)	277	16.9 (1.5)	272	16.8 (1.5)
2.5 years	401	16.8 (1.4)	203	16.9 (1.4)	198	16.7 (1.4)
3.0 years	148	16.7 (1.9)	74	16.7 (1.9)	74	16.8 (1.9)
BMI z-score (SD)^5^	549	0.47 (1.0)	277	0.51 (1.0)	272	0.43 (1.0)
BMI classification (%)^6^						
Thinness (I, II)	32	5.8	14	5.1	18	6.6
Normal weight	404	73.6	200	72.2	204	75.0
Overweight	88	16.0	50	18.0	38	14.0
Obesity (I, II)	25	4.6	13	4.7	12	4.4
Vegetables and fruit/berries (g/day)	541	214.7 (74.8)	270	220.3 (74.5)	271	209.2 (74.9)
Vegetables (g/day)	541	43.7 (19.4)	270	46.2 (18.7)	271	41.1 (19.7)
Fruit/berries (g/day)	541	171.1 (64.8)	270	174.1 (65.1)	271	168.0 (64.5)
Sweet and savory treats (g/day)	541	24.4 (26.0)	270	21.9 (24.1)	271	26.8 (27.5)
Sweet drinks (g/day)	543	87.7 (124.1)	271	82.5 (127.6)	272	92.8 (120.5)
MVPA (min/day)	539	117.7 (57.0)	269	114.9 (58.2)	270	120.4 (55.9)
MVPA, weekday (min/day)	541	118.7 (61.2)	270	116.2 (62.6)	271	121.2 (59.8)
MVPA, weekend (min/day)	540	114.9 (57.2)	270	112.0 (57.3)	270	117.8 (57.1)
Screen time (min/day)	541	70.0 (39.7)	270	67.2 (39.1)	271	72.8 (40.1)
Screen time, weekday (min/day)	542	62.3 (39.2)	270	59.8 (38.4)	272	64.9 (39.8)
Screen time, weekend (min/day)	541	89.6 (49.1)	270	85.9 (49.7)	271	93.2 (48.3)

*Abbreviations* *CI* confidence interval, *MVPA* moderate-to-vigorous physical activity, *PSE* parental self-efficcy *M* Mean, *SD* Standard deviation, *PSE* parental self-efficacy, *BMI* Body Mass Index, *MVPA* moderate-to-vigorous physical activity

^1^Characteristics presented as percentages (%) or as mean and standard deviation (M ± SD)

^2^Characteristics of the participating parent, i.e., the parent that filled out the baseline questionnaire and activated and used the MINISTOP 2.0 app on their mobile phone, if randomized to the intervention group

^3^Mean PSE score for promoting healthy lifestyle behaviors (diet, physical activity, screen time). Score range for each question: 1–10 [34]

^4^When considering country of birth for the whole family (i.e., also parents), 61.2% of children had parents that were both born in Sweden, 23.6% had two foreign-born parents, and 15.2% had one parent that was born in Sweden and one foreign-born parent

^5^BMI standard deviation scores (BMI z-scores) calculated using the extended international age and sex specific body mass index (IOTF) cut-offs by Cole and Lobstein 2012 [43]

^6^BMI classification according to Cole and Lobsteins revised cut-offs 2012 [43]: Thinness II, ISO-BMI < 17.0 kg/m^2^; Thinness I, ISO-BMI = 17.0–18.5 kg/m^2^; Normal weight, ISO-BMI = 18.5–24.9 kg/m^2^; Overweight, ISO-BMI = 25.0–29.9 kg/m^2^; Obesity I, ISO-BMI = 30.0–34.9 kg/m^2^; Obesity II, ISO-BMI = 35.0–39.9 kg/m^2^

### Effectiveness of the intervention

#### Primary outcomes

**Fig. 3:**
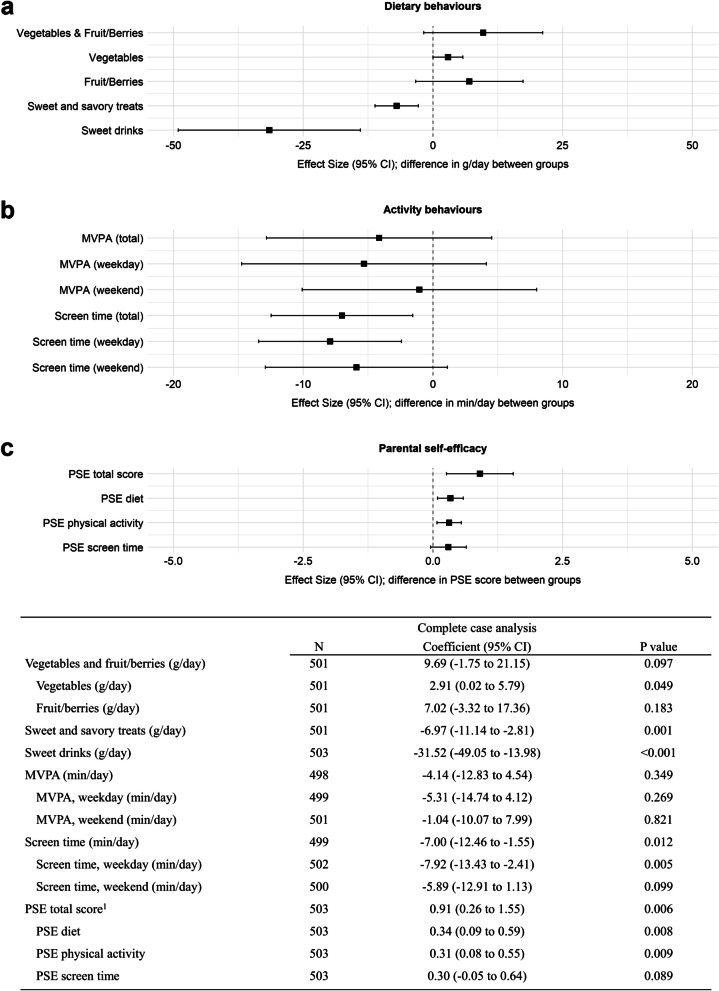
Results from the complete case analysis (*n* = 503) showing the effect of the intervention on primary and secondary outcomes at follow-up. The effect of the intervention on primary outcomes is shown in a) vegetables and fruit/berries, sweet and savory treats, and sweet drinks (g/day), and in b) moderate-to-vigorous physical activity (MVPA) and screen time (min/day) whereas c) shows the effect of the intervention on the secondary outcome parental self-efficacy (PSE) for promoting healthy lifestyle behaviors at follow-up. All models were adjusted for the respective baseline outcome, the child’s sex and age at baseline, and random intercepts were added for child health care center site Abbreviations: *CI* Confidence interval, *MVPA* Moderate-to-vigorous physical activity, *PSE* Parental self-efficacy. ^1^Mean PSE score for promoting healthy lifestyle behaviors (diet, physical activity, screen time). Score range for each question: 1-10 [34]

The effect of the intervention on primary outcomes are presented in Fig. 3. We observed statistically significant intervention effects on mean intakes of sweet and savory treats (-6.97 g/day; 95% CI -11.14 to -2.81; *p* = 0.001), sweet drinks (-31.52 g/day; 95% CI -49.05 to -13.98; *p* < 0.001) and average time spent in front of a screen (-7.00 min/day; 95% CI -12.46 to -1.55; *p* = 0.012) at follow-up. We found no statistically significant effect on MVPA (-4.14 min/day; 95% CI -12.83 to 4.54; *p* = 0.349) or the intake of vegetables and fruits/berries at follow-up (9.69 g/day; 95% CI -1.75 to 21.15; *p* = 0.097). However, when analyzing intakes of vegetables and fruits/berries separately we observed a small yet statistically significant effect on the intake of vegetables (2.91 g/day; 95% CI 0.02 to 5.79; *p* = 0.049). We found no evidence that the intervention effects were moderated by parental education or country of birth, except for an interaction between parental country of birth and group allocation, with the intervention being more effective for intake of sweet and savory treats (-10.90 g/day; 95% CI -21.00 to -0.79; *p* = 0.036) when both parents were born outside of Sweden.

The effect of the intervention on the primary outcomes was further supported by Bayesian analyses (Supplementary information, Figure S2) where the probability of the intervention having any effect on the intake of fruit and vegetables compared to the control group was 94.6%. Likewise, the probability of the intervention having any effect on intakes of sweet and savory treats and sweet drinks was 99.9% and ≥ 99.9%, respectively. For MVPA and screen time, the probability of an intervention effect was 82.4% and 99.3% respectively.

### Secondary outcomes

The effects of the intervention on PSE for promoting healthy lifestyle behaviors is also presented in Fig. 3. Parents in the intervention group reported a significantly higher total PSE score (0.91; 95% CI 0.26 to 1.55; *p* = 0.006) at follow-up compared to the control group. The effect was driven by the PSE score for promoting healthy diet (0.34; 95% CI 0.09 to 0.59; *p* = 0.008) and the PSE score for promoting healthy physical activity behaviors (0.31; 95% CI 0.08 to 0.55; *p* = 0.009). In terms of children’s BMI z-score, quantile regression analyses revealed no statistically significant effect at follow-up (50^th^ percentile: 0.0; 95% CI -0.09 to 0.09; and 90^th^ percentile: 0.04; 95% CI -0.03 to 0.11; both *p* > 0.05).

### Attrition and sensitivity analyses

There were no marked associations between baseline characteristics and missing data at follow-up. This was the case when modelling baseline characteristics versus the reason for follow-up data being missing, as well as when modelling missingness for each primary outcome separately. Findings from both normal regression and Bayesian analyses with missing data imputed were not different than findings from complete case analyses for all outcomes (Supplementary information, Table S1).

### Process evaluation

Self-reported data on app usage and satisfaction (*n* = 154) are presented in Table 2. In summary, 79% of the parents fully agreed to somewhat agreed that they were satisfied with the app. On the questions whether the app had given them insight into their child’s diet and physical activity behaviors and whether the app had supported them in creating healthy lifestyle behaviors, 76% and 65% of parents respectively answered that they fully agreed to somewhat agreed. Fifty-four percent of parents reported to have used the app once a week or more and 67% reported to have taken part in the majority of the themes (≥ 7–8 themes). Furthermore, objective analytics data on parental engagement with the registration feature in the MINISTOP app showed that parents entered their child’s health behaviors on average once per week during the 6-month intervention period for all four categories (*n* = 277): 1) MVPA and screen time (1.05 ± 1.95 days per week); 2) sweet drinks (1.01 ± 1.95 days per week); 3) sweet and savory treats (1.01 ± 1.93 days per week); and 4) fruit and vegetables (1.08 ± 1.97 days per week).

**Table 2:** Participating parents’ (*n* = 154) self-reported satisfaction and usage of the MINISTOP 2.0 app

	Fully agree	Agree		Agree to some extent	Disagree		Strongly disagree		Do not know
Questions, acceptability:	*n* (%)	*n* (%)		*n* (%)	*n* (%)		*n* (%)		*n* (%)
I am satisfied with the app	31 (20.1)	56 (36.4)		35 (22.7)	13 (8.4)		8 (5.2)		11 (7.1)
The app has given me insight into what my child’s diet and activity habits look like	35 (22.7)	42 (27.3)		40 (26.0)	16 (10.4)		5 (3.2)		16 (10.4)
The app has supported me in creating healthy diet and activity habits for my child	31 (20.1)	34 (22.1)		35 (22.7)	22 (14.3)		11 (7.1)		21 (13.6)
It was easy to understand the features in the app	83 (53.9)	48 (31.2)		8 (5.2)	3 (1.9)		1 (0.6)		11 (7.1)
I perceived the content in the app as factually correct	82 (53.2)	52 (33.8)		5 (3.2)	1 (0.6)		0 (0.0)		14 (9.1)
I found the diet and activity registration feature in the app helpful	36 (23.4)	34 (22.1)		33 (21.4)	15 (9.7)		18 (11.7)		18 (11.7)
I received useful tips/information from the messages/push notifications	28 (18.2)	49 (31.8)		36 (23.4)	13 (8.4)		8 (5.2)		20 (12.9)
I would recommend other parents to use the MINISTOP 2.0 app	30 (19.5)	40 (26.0)		35 (22.7)	18 (11.7)		9 (5.8)		22 (14.3)
	Everyday	More than three times per week	Two–three times per week	Once weekly	Two–three times per month	Once per month		Less than once per month	Never
Questions, app usage^1^:	n (%)	n (%)	n (%)	n (%)	n (%)	n (%)		n (%)	n (%)
How actively did you use the app?	3 (2.2)	31 (22.3)	17 (12.2)	24 (17.3)	21 (15.1)	14 (10.1)		25 (18.0)	4 (2.9)
	All 13 themes	11–12 themes	9–10 themes	7–8 themes	5–6 themes	3–4 themes		1–2 themes	None
	n (%)	n (%)	n (%)	n (%)	n (%)	n (%)		n (%)	n (%)
How many themes did you take part of?^2^	38 (27.3)	14 (10.1)	18 (12.9)	23 (16.5)	22 (15.8)	13 (9.4)		5 (3.6)	6 (4.3)

^1^*n* = 139

^2^The MINISTOP 2.0 app included 13 themes with information, practical tips, and strategies for promoting healthy diet, physical activity, screen time, sleep and dental care behaviors for children aged 2–3 years

## Discussion

### Main findings

This study is the first of its kind to report real-world effectiveness of a parent-oriented mHealth intervention in 2.5-to-3-year-old children, delivered through primary child health care, with the overall aim to promote healthy lifestyle behaviors. An intervention effect was observed, with children in the intervention group having lower reported intakes of sweet and savory treats, sweet drinks, as well as less screen time at follow-up compared to the control group (primary outcomes). Additionally, parents in the intervention group also reported a higher PSE score for promoting healthy lifestyle behaviors at follow-up than the control group (secondary outcomes).

### Comparison with previous work

To date, there are no other app-based interventions targeting healthy lifestyle behaviors in preschool-aged children offered through child health care. However, a few web-based eHealth interventions have been conducted, that were also linked to a child/youth health care setting. Van Grieken et al*.* [46] observed small improvements in health-related behaviors (e.g., sweet drinks and screen time) when analyzing subgroups of children; however, no effects were observed for the entire group when evaluating web-based personalized advice offered to parents (*n* = 2102) prior to health youth center visits at either 18- or 24-months of age. Helle et al*.* (2019) [47] found that children in the intervention group were more frequently served vegetables and fruits (*p* = 0.015) and less likely to have screen time whilst eating (*p* = 0.009) after a 6-month intervention of monthly videos promoting healthy eating behaviors, to parents of 6-month-year-old children (*n* = 718). Similar to these interventions, we observed significant effects on lifestyle behaviors such as intake of sweet and savory treats, sweet drinks, and screen time, indicating promising future potential for digital interventions, when incorporated or linked to child health care systems.

We also observed a statistically significant effect on screen time. This effect was possibly driven by screen time during weekdays, where an effect size of almost 8 min per day less screen time was observed. Interestingly, this effect is comparable to the long-term follow-up results of the INFANT trial: a more traditional and resource-intense face-to-face intervention delivered by dietitians in 2-h sessions every three months to first time parents of infants aged 4 months [48]. Briefly, parents in the intervention group reported 10 min/day less screen viewing at both follow-ups (2- and 3.5-years post-intervention, i.e., at 3.6 and 5 years respectively) [48]. We did not observe any difference in MVPA between groups at follow-up, which may be due to the primary focus on dietary behaviors in the MINISTOP 2.0 app themes. Also, movement patterns of children aged 2-to-3 years are quite different compared to older children as they are often characterized by an intermittent movement pattern and activities that are not structured or planned [49] making it harder for parents to identify changes in MVPA even though they may have occurred.

Interestingly, parents in the intervention group reported a higher PSE for promoting healthy diet and physical activity behaviors at follow-up. As parents are key players in promoting healthy lifestyle behaviors for this age group, we believe that this was a meaningful finding. Our results are also in accordance with Möhler et al*.* [50] where PSE was a positive predictor of children’s intakes of fruits and vegetables in a kindergarten based randomized controlled trial (*n* = 558). Correspondingly, a child-centered health dialogue (i.e., a face-to-face intervention) at the age of 4 years within Swedish primary child health care [51] resulted in a positive effect on maternal PSE for promoting healthy physical activity behaviors. Altogether, it is reasonable to conclude that our findings and existing literature suggest that PSE may be an important target for health promotion in preschool-aged children.

We did not observe any intervention effect on children’s BMI z-score, however, this was not expected as it may take longer than six months to observe changes in children’s body weight. Furthermore, the intervention was primarily aimed at promoting healthy lifestyle behaviors and therefore included children from all BMI-categories, with the majority classified as having normal weight. Due to the above-mentioned reasons, BMI was also defined as our secondary outcome a priori. Still, the positive intervention effects on dietary and screen time behaviors and PSE, could lead to potential positive effects on BMI later on in childhood. Our null finding for BMI is also in line with the results of Derwig et al*.* [52], where no statistically significant effect on BMI-z score in Swedish 4-year-olds was obtained at follow-up after their health care-based intervention. Correspondingly, van Grieken et al*.* did not observe any effects on children’s BMI z-score at the follow-up at 36 months of age, after the parents had received web-based information on healthy lifestyle behaviors [46]. Finally, the purpose of the MINISTOP 2.0 intervention is prevention of childhood obesity and not treatment. As childhood obesity is a complex multifactorial disease, successful treatment, i.e., a statistically significant decrease in BMI (≥ 0.25 BMI z-score) may require more intense face-to-face health care-based efforts in high-risk populations, where an app like MINISTOP 2.0 could be used as a complementary support tool between visits.

### Generalizability, strengths, and limitations

This was an effectiveness trial with the intention of being as close to real-world circumstances as possible, within the limits of necessary trial procedures. As families were recruited from 19 child health care centers in six Swedish regions, covering various geographic and socioeconomic contexts, the generalizability of our findings is high in comparison to previously conducted efficacy trials. We also translated and adapted the app into Somali, Arabic and English to reach as many families in the Swedish population as possible. Our efforts were successful; compared to the first MINISTOP trial, where only 9% of children had parents born outside Sweden [24], 24% of children had two foreign-born parents in the present trial. This is very similar to the Swedish general population, where approximately 24% of children have parents born in another country [26]. It is also relevant to note that among the participating foreign-born parents, 51% were from North Africa, Sub-Saharan Africa, and the Middle East, which currently are among the most common languages spoken after Swedish (i.e., Arabic and Somali). Together with the low attrition rate (9%), this further strengthens the generalizability of our findings. Other major strengths of the trial are that the development of the MINISTOP intervention (1.0 and 2.0) was guided by theory [36, 37] and the overarching hybrid type 1 effectiveness-implementation design. The hybrid type 1 design enables exploration of implementation aspects through qualitative interviews with families, health care professionals and stakeholders to facilitate the future implementation of the intervention within primary child health care [29]. These results will be reported separately and will provide a valuable complement to the present study results as intervention effectiveness alone does not ensure future implementation success and adoption within the intended setting [53, 54].

The study also has some limitations. Since the nurses provided access to the app, they were not blinded to the group allocation which may be a limitation as it introduces the possibility of nurses providing additional information on healthy eating and physical activity to the control group participants knowing they would not receive the app. This may dilute the true effect, however, all nurses were pre-trained in the study protocol by the research team and hence we consider this risk as unlikely. Also, data imputation, processing, and analyses were conducted by the research team. Thus, we do not consider that non-blinded assessors had any major influence on our findings. Another limitation was the use of self-reported outcome data, which may be subject to recall bias but also social desirability bias as parents were not anonymous when reporting their child’s health behaviors in the questionnaire, and thus may have reported healthier behaviors than they actually had. Furthermore, even though a well-established and validated questionnaire was used [33], it has not been specifically evaluated for this age group which is a further limitation. However, the use of self-report was necessary as we wanted to evaluate the intervention within child health care, and more objective outcome measures would have made the data collection too burdensome for the nurses. As we have previously evaluated MINISTOP using accurate and objective outcome measures [24], the focus and novelty of this trial was the evaluation within the primary child health care setting. Notably, we were also able to replicate our findings from the first efficacy trial with regards to the intervention effect on health behaviors, using outcome measures that were more feasible for the child health care setting. In the first trial, the intervention group had higher odds of increasing a composite score for six dietary or activity behaviors (OR; 1.99; 95% CI: 1.20, 3.30; *P* = 0.008) compared to the control group [24], with lower mean intake of sweet drinks (-12 g/day versus + 8 g/day; *p* = 0.049) at follow-up.

### Clinical and public health relevance

Although the effect size for sweet and savory treats was relatively small (-6.97 g/day, *p* = 0.001) we observed a larger intervention effect on sweet drinks (-31.52 g/day, *p* < 0.001). There is no national data on daily intakes of sweet drinks for children aged 2.5–3 years, however, for comparison, a decrease in 32 g would correspond to a decrease by 20% of total reported daily intakes of sweet drinks in Swedish 4-year-olds [55]. Furthermore, we observed a slightly higher PSE in the intervention group compared to the control at follow-up. This result was further supported by self-reported data on app satisfaction, where a large proportion of parents stated that the app had made them more aware of their child’s diet and physical activity behaviors, and that the app had supported them in creating healthier lifestyle behaviors. A high proportion of the parents also reported to have used the app regularly, which was supported by objective usage data on the registration feature. Also, as the MINISTOP 2.0 is an app-based intervention, it requires very little effort from health care professionals. Finally, our qualitative work from interviews with parents and child health care nurses reveal high satisfaction and engagement with the app (results will be reported separately). To conclude, these real-world effectiveness results, together with the results of our first efficacy trial (MINISTOP 1.0) [24] provide further support for the MINISTOP app as a strategy for promoting healthy lifestyle behaviors in preschool-aged children. Thus, future steps include actions for implementation of the app within primary child health care at scale [54], including assessments of cost-effectiveness.

## Conclusions

This study reports the real-world effectiveness of the MINISTOP 2.0 app in a population-based sample, where 24% of the children had two foreign-born parents. Overall, the intervention resulted in statistically significant lower intakes of sweet and savory treats, sweet drinks, and screen time, as well as higher PSE when compared to the control group. Altogether, these findings together with the results from our previous efficacy trial (MINISTOP 1.0) support the implementation of the MINISTOP 2.0 intervention within primary child health care at scale.

## Supplementary Information

**Additional file 1: Figure S1.** Program theory illustrating how the MINISTOP 2.0 intervention is grounded in social cognitive theory and various behavior change techniques to increase parental knowledge, skills, and self-efficacy to support and enable behavior change for improved diet and physical activity behaviors in children.

**Additional file 2: Figure S2**. Results from the Bayesian analysis of complete cases (*n*=503). The graphs show marginal posterior distributions for estimates of effects on primary outcomes: a) vegetables and fruit/berries, b) sweet and savory treats, c) sweet drinks, d) moderate-to-vigorous physical activity (MVPA) and e) screen time.

**Additional file 3: Table S1.** Results from the imputed data analyses (*n* =552) using linear regression and Bayesian analysis respectively. All regression models (primary and secondary outcomes) were adjusted for the respective baseline outcome, the child’s sex and age at baseline, and random intercepts were added for child health care center site.

## Data Availability

The datasets created and analyzed during the present study are available from the corresponding author upon reasonable request.

## Abbreviations

mHealth: Mobile health
App: Application
BMI: Body mass index
BMI z-score: Body mass index standard deviation score
SD: Standard deviation
