# Supplementary material for: Effectiveness of a Smartphone App (MINISTOP 2.0) integrated in primary child health care to promote healthy diet and physical activity behaviors and prevent obesity in preschool-aged children: randomized controlled trial
Source: Int J Behav Nutr Phys Act. 2023 Feb 21;20:22. doi: 10.1186/s12966-023-01405-5 (PMC9942425; doi:10.1186/s12966-023-01405-5)
Supplement: Supplementary file 1 — Additional file 1: Figure S1. Program theory illustrating how the MINISTOP 2.0 intervention is grounded in social cognitive theory and various behavior change techniques to increase parental knowledge, skills, and self-efficacy to support and enable behavior change for improved diet and physical activity behaviors in children. [file 12966_2023_1405_MOESM1_ESM.docx]

| **Figure S1.** Program theory illustrating how the MINISTOP 2.0 intervention is grounded in social cognitive theory and various behavior change techniques to increase parental knowledge, skills, and self-efficacy to support and enable behavior change for improved diet and physical activity behaviors in children. |
| --- |
| 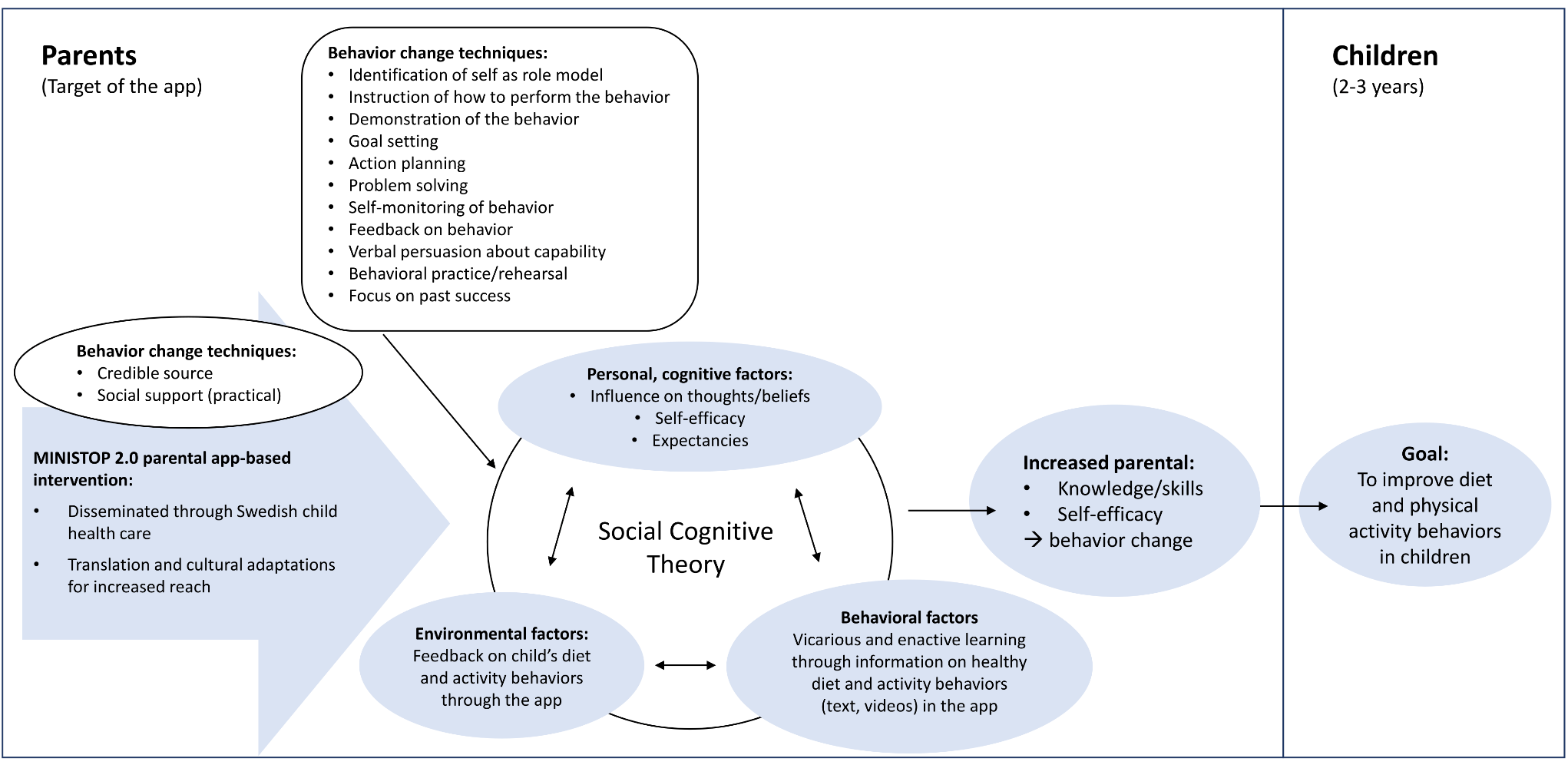 |
