# Supplementary material for: Effectiveness of a Smartphone App (MINISTOP 2.0) integrated in primary child health care to promote healthy diet and physical activity behaviors and prevent obesity in preschool-aged children: randomized controlled trial
Source: Int J Behav Nutr Phys Act. 2023 Feb 21;20:22. doi: 10.1186/s12966-023-01405-5 (PMC9942425; doi:10.1186/s12966-023-01405-5)
Supplement: Supplementary file 2 — Additional file 2: Figure S2. Results from the Bayesian analysis of complete cases (n=503). The graphs show marginal posterior distributions for estimates of effects on primary outcomes: a) vegetables and fruit/berries, b) sweet and savory treats, c) sweet drinks, d) moderate-to-vigorous physical activity (MVPA) and e) screen time. [file 12966_2023_1405_MOESM2_ESM.docx]

| **Figure S2.** Results from the Bayesian analysis of complete cases (n=503). The graphs show marginal posterior distributions for estimates of effects on primary outcomes: a) vegetables and fruit/berries, b) sweet and savory treats, c) sweet drinks, d) moderate-to-vigorous physical activity (MVPA) and e) screen time. |
| --- |
| 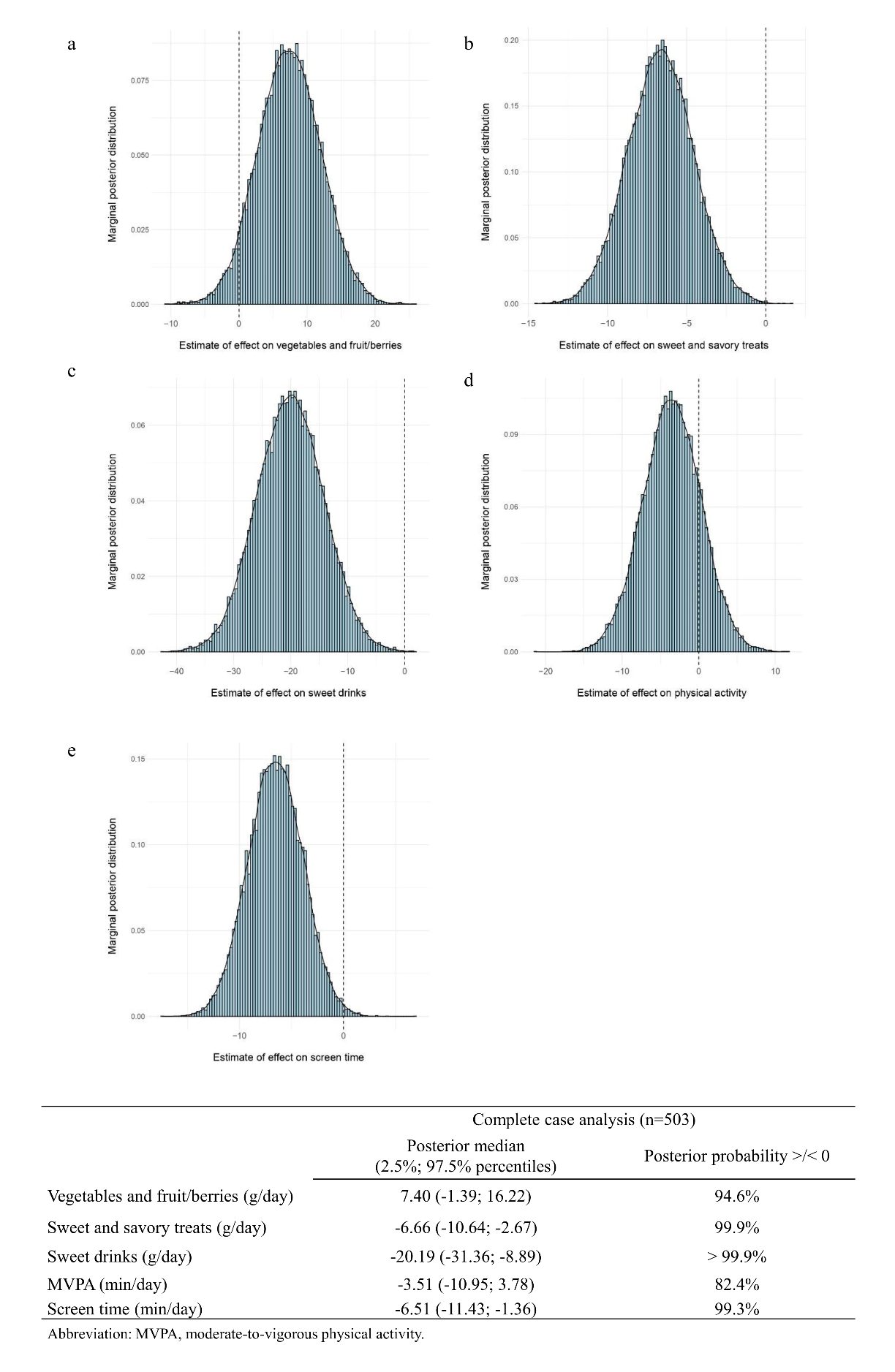 |
