# Supplementary material for: Effectiveness of a Smartphone App (MINISTOP 2.0) integrated in primary child health care to promote healthy diet and physical activity behaviors and prevent obesity in preschool-aged children: randomized controlled trial
Source: Int J Behav Nutr Phys Act. 2023 Feb 21;20:22. doi: 10.1186/s12966-023-01405-5 (PMC9942425; doi:10.1186/s12966-023-01405-5)
Supplement: Supplementary file 3 — Additional file 3: Table S1. Results from the imputed data analyses (n =552) using linear regression and Bayesian analysis respectively. All regression models (primary and secondary outcomes) were adjusted for the respective baseline outcome, the child’s sex and age at baseline, and random intercepts were added for child health care center site. [file 12966_2023_1405_MOESM3_ESM.docx]

| **Table S1.** Results from the imputed data analyses (n=552) using linear regression and Bayesian analysis respectively. All regression models (primary and secondary outcomes) were adjusted for the respective baseline outcome, the child’s sex and age at baseline, and random intercepts were added for child health care center site. | | | | | | | |
| --- | --- | --- | --- | --- | --- | --- | --- |
|  | **Normal regression** | | | | | **Bayesian analysis** | |
|  | Imputed data analysis | | | | | Imputed data analysis | |
| **Primary outcomes** | N | Coefficient (95% CI) | P value |  | N | Posterior median (2.5%; 97.5% percentiles) | Posterior probability >/< 0 |
| Vegetables and fruit/berries (g/day) | 552 | 10.01 (-1.39 to 21.40) | 0.085 |  | 552 | 8.01 (-1.08; 17.16) | 95.8% |
| Vegetables (g/day) | 552 | 3.09 (0.20 to 5.98) | 0.036 |  | 552 | 3.01 (0.21; 5.82) | 98.2% |
| Fruit/berries (g/day) | 552 | 7.06 (-3.23 to 17.35) | 0.178 |  | 552 | 5.90 (-2.59; 14.44) | 91.3% |
| Sweet and savory treats (g/day) | 552 | -6.48 (-10.76 to -2.20) | 0.003 |  | 552 | -6.16 (-10.24; -2.08) | 99.8% |
| Sweet drinks (g/day) | 552 | -31.89 (-49.54 to -14.23) | <0.001 |  | 552 | -21.03 (-32.88; -9.22) | >99.9% |
| MVPA (min/day) | 552 | -3.78 (-12.37 to 4.81) | 0.388 |  | 552 | -3.15 (-10.66; 4.35) | 79.4% |
| MVPA, weekday (min/day) | 552 | -5.24 (-14.57 to 4.09) | 0.270 |  | 552 | -4.27 (-12.26; 3.74) | 85.3% |
| MVPA, weekend (min/day) | 552 | -0.33 (-9.35 to 8.69) | 0.943 |  | 552 | -0.39 (-8.14; 7.38) | 53.9% |
| Screen time (min/day) | 552 | -7.33 (-12.77 to -1.90) | 0.008 |  | 552 | -6.92 (-12.04; -1.76) | 99.6% |
| Screen time, weekday (min/day) | 552 | -8.00 (-13.45 to -2.55) | 0.004 |  | 552 | -7.53 (-12.65; -2.41) | 99.8% |
| Screen time, weekend (min/day) | 552 | -6.36 (-13.44 to 0.73) | 0.079 |  | 552 | -5.78 (-12.13; 0.59) | 96.2% |
| **Secondary outcomes** |  |  |  |  |  |  |  |
| PSE total score^1^ | 552 | 0.88 (0.23 to 1.52) | 0.008 |  | 552 | 0.86 (0.21; 1.5) | 99.5% |
| PSE diet | 552 | 0.32 (0.06 to 0.57) | 0.014 |  | 552 | 0.31 (0.06; 0.56) | 99.2% |
| PSE physical activity | 552 | 0.32 (0.09 to 0.56) | 0.007 |  | 552 | 0.32 (0.08; 0.55) | 99.6% |
| PSE screen time | 552 | 0.27 (-0.07 to 0.62) | 0.115 |  | 552 | 0.27 (-0.07; 0.62) | 94.0% |
| Abbreviations: CI, confidence interval; MVPA, moderate-to-vigorous physical activity; PSE, parental self-efficacy.  ^1^Mean PSE score for promoting healthy lifestyle behaviors (diet, physical activity, screen time). Score range for each question: 1-10 [34]. | | | | | | | |
